# Suppressing PD-L1 Expression via AURKA Kinase Inhibition Enhances Natural Killer Cell-Mediated Cytotoxicity against Glioblastoma

**DOI:** 10.3390/cells13131155

**Published:** 2024-07-06

**Authors:** Trang T. T. Nguyen, Qiuqiang Gao, Jeong-Yeon Mun, Zhe Zhu, Chang Shu, Aaron Naim, Meri Rogava, Benjamin Izar, Mike-Andrew Westhoff, Georg Karpel-Massler, Markus D. Siegelin

**Affiliations:** 1 Department of Pathology and Cell Biology, Columbia University Medical Center, New York, NY 10032, USA; thithutrang.nguyen@nyulangone.org (T.T.T.N.); qg2170@cumc.columbia.edu (Q.G.); jm5576@cumc.columbia.edu (J.-Y.M.); zz2836@cumc.columbia.edu (Z.Z.); cs485@cumc.columbia.edu (C.S.); abn2137@cumc.columbia.edu (A.N.); 2 Division of Hematology/Oncology and Herbert Irving Comprehensive Cancer Center, Columbia University Medical Center, New York, NY 10032, USA; mr4020@cumc.columbia.edu (M.R.); bi2175@cumc.columbia.edu (B.I.); 3 Department of Pediatrics and Adolescent Medicine, Ulm University Medical Center, 89081 Ulm, Germany; andrew.westhoff@web.de; 4 Department of Neurosurgery, Ulm University Medical Center, 89081 Ulm, Germany; georg.karpel@gmail.com

**Keywords:** Aurora kinase A, PD-L1, glioblastoma, NK-cells

## Abstract

Immunotherapies have shown significant promise as an impactful strategy in cancer treatment. However, in glioblastoma multiforme (GBM), the most prevalent primary brain tumor in adults, these therapies have demonstrated lower efficacy than initially anticipated. Consequently, there is an urgent need for strategies to enhance the effectiveness of immune treatments. AURKA has been identified as a potential drug target for GBM treatment. An analysis of the GBM cell transcriptome following AURKA inhibition revealed a potential influence on the immune system. Our research revealed that AURKA influenced PD-L1 levels in various GBM model systems in vitro and in vivo. Disrupting AURKA function genetically led to reduced PD-L1 levels and increased MHC-I expression in both established and patient-derived xenograft GBM cultures. This process involved both transcriptional and non-transcriptional pathways, partly implicating GSK3β. Interfering with AURKA also enhanced NK-cell-mediated elimination of GBM by reducing PD-L1 expression, as evidenced in rescue experiments. Furthermore, using a mouse model that mimics GBM with patient-derived cells demonstrated that Alisertib decreased PD-L1 expression in living organisms. Combination therapy involving anti-PD-1 treatment and Alisertib significantly prolonged overall survival compared to vehicle treatment. These findings suggest that targeting AURKA could have therapeutic implications for modulating the immune environment within GBM cells.

## 1. Introduction

Aurora kinase A, also known as serine/threonine-protein kinase 6, encoded by gene AURKA, a crucial regulator of the cell cycle, plays a significant role in cancer progression [1,2]. As a result, it has emerged as a promising target for cancer treatment. Several inhibitors have been developed to target AURKA and have progressed to clinical trials. One such inhibitor is Alisertib, which has undergone clinical testing in various types of cancer. In estrogen-positive breast cancer, amplification of AURKA is linked to resistance against cyclin-dependent kinase 4/6 inhibitor (CDK 4/6i) [3]. Consequently, interference with AURKA leads to an increase in hormone receptors and enhances the sensitivity of breast cancer cells to endocrine therapy. However, recent clinical trials showed limited effectiveness of Alisertib in metastatic breast cancer patients who were resistant to CDK4/6i and endocrine therapy [3]. In glioblastoma (GBM), the most common primary brain tumor in adults with a poor prognosis of 12–18 months [4,5,6,7,8,9,10,11,12,13], Alisertib demonstrated preclinical activity in orthotopic GBM models that led to extended survival in animal subjects [1]. Furthermore, there was evidence that Alisertib exhibited anti-glioma activity even in GBM model systems that were resistant to bevacizumab therapy, a monoclonal antibody that binds to VEGF protein from cancer cells and blocks blood vessel growth [1]. Similarly, our research found that Alisertib also displayed anti-glioma activity within specific Patient-Derived Xenograft (PDX) GBM model systems [14]. This previous research indicates that AURKA has the potential to be a promising choice for combination treatments.

Therapeutic responses to AURKA blockage appear to be partly dependent on transcription factor c-Myc [15]. High levels of this protein have been linked to predicting therapeutic responses [15]. In the context of GBM, it has been found that c-Myc interacts with AURKA, which in turn stabilizes it and prevents proteasome-mediated degradation [14]. These effects seem to be at least partially reliant on protein kinase GSK3β [14]. Regarding N-Myc, a factor often highly upregulated in neuroblastoma and sharing overlapping targets with c-Myc, research has shown that AURKA also stabilizes N-Myc [16]. Neuroblastoma cells with high levels of N-Myc appeared more susceptible to Alisertib treatment [16].

Immune cell therapies for solid tumors and GBM are rapidly advancing, involving various strategies such as the use of chimeric antigen receptors-T-cells, natural killer (NK)-cells, and modulation of the immunosuppressive microenvironment (e.g., myeloid-derived suppressor cells) [17,18,19]. NK cells form a crucial part of the body’s innate immune system, responsible for eliminating infected or cancerous cells. Unlike T-cells, NK cells do not need specific activation by antigens and can, therefore, target cancer cells spontaneously. Advancements in molecular engineering have resulted in the creation of chimeric antigen receptor NK cells, allowing them identification of antigens like oncogenic surface growth factors such as HER2 and EGFR. These engineered NK cells are being used for therapeutic purposes.

The programmed cell death protein 1 (PD-1) pathway has garnered attention because previous research demonstrated a significant decrease in tumor growth in PD-1 knockout mice compared to wild-type mice [20]. Therapeutic antibodies targeting PD-1 reinvigorate the immune cell (mostly T cell)-mediated destruction of cancer cells [21]. Earlier studies also emphasized the significance of the PD-1 pathway in GBM [22]. The scientific community is actively working on enhancing immune cell-mediated killing of GBM cells. Based on existing literature, combining immune cell-based therapies with other treatments may potentially benefit some GBM patients, although further research is needed to fully evaluate this approach. Such combination therapies may impact different aspects of the immune system, including modulating the levels of immune checkpoint inhibitors.

NK-92 cells are derived from a patient with non-Hodgkin’s lymphoma [23,24]. It is intriguing to note that these cells have been employed for the treatment of cancer in patients due to their observed killing activity against various cancer cells, including solid and hematological tumors [23,24,25]. This method of adoptive transfer, which utilizes NK-92 cells, necessitates irradiation beforehand to prevent these cells from multiplying within the patients [26]. Furthermore, they have also been engineered to become CAR-NK-92 against a variety of different targets, such as HER2 and others [23,27,28]. Upon co-cultivation with tumor cells, it has been experimentally demonstrated that NK cells upregulate the expression of PD-1 on their surface [29]. This versatility makes them promising candidates for targeted cancer therapy.

Our research reveals that AURKA plays a role in regulating the ability of NK cells to target and eliminate GBM cells. This regulation is partially achieved by influencing PD-L1, which is a key target for medical interventions such as immune checkpoint inhibitors. Additionally, we observe that disrupting AURKA pharmacologically can boost the effectiveness of immune checkpoint inhibitor therapy in a GBM syngeneic model.

## 2. Materials and Methods

### 2.1. Cell Cultures

PDX GBM22 and GBM12 cells were sourced from Dr. Jann Sarkaria at Mayo Clinic between 2020 and 2023. The molecular subtype of GBM22 is classified as classical with EGFR gain, TP53 mutation, and MYC amplification, while the molecular subtype of GBM12 is mesenchymal with EGFR amplification and TP53 mutation (splice mutation). U251 cells were obtained from Sigma (Catalog #09063001-1VL), while GL261 cells were acquired from Repository of Tumors and Tumor Cell Lines at NCI in Bethesda, MD. The U87 cell line came from the American Type Culture Collection (Catalog #HTB-14), while the U87 EGFRvIII cell line was supplied by Frank Furnari at UCSD in La Jolla, California. NK-92 MI cells, known for their continuous expression of interleukin-2, were procured from the ATCC (Catalog #CRL-2408).

GBM22, GBM12, U87, U251, and U87 EGFRvIII cells were sustained in DMEM (Fisher Scientific, MT10013CV) with 10% FBS (GeminiBio (West Sacramento, CA, USA), 100–500) and Primocin (Invivogen (San Diego, CA, USA), ant-pm-1) at a concentration of 100 μg/mL. Cells were cultured in DMEM supplemented with 1.5% FBS and 100 μg/mL of primocin for the specific drug treatment. The NCH644 cell line, which exhibits characteristics of human stem cells, was acquired from Cell Line Services (Cat # 820403) and maintained in StemPro NSC SFM (Thermo Fisher, A1050901) supplemented with 100 µg/mL primocin for both regular culture maintenance and drug treatment. Astrocyte cells were sourced from ScienceCell Research Laboratories in Carlsbad, CA. They were then grown in a culture medium comprising DMEM, 10% FBS, primocin, and N2 supplement (Catalog #17502048) obtained from Thermo Fisher. NK-92 MI cells were cultured in MyeloCult™ H5100 media (Catalog #05150) containing Hydrocortisone (Catalog #74142). Alisertib-resistant GBM22 cell lines were developed through prolonged exposure to Alisertib for a duration of two weeks. The cells were cultured and kept at a temperature of 37 °C with 5% CO_2_.

### 2.2. Reagents

Alisertib (MLN8237) and BMS-1166 (HY-102011) were purchased from MedChemExpress. MG-132 (474791) and puromycin dihydrochloride (P9620) were acquired from Sigma. CHIR-99021 (Catalog #4423) was purchased from (TOCRIS).

### 2.3. Cell Viability Assays

GBM cells were exposed to medications for a period of 72 h, and cell viability assessments were conducted using the CellTiter-Glo assays from Promega (G7571) or Crystal Violet Assay Kit (Abcam (Waltham, MA, USA), ab232855) in accordance with the manufacturer’s guidelines.

### 2.4. Flow Cytometry

GBM cells were treated with drugs for 24 h and were stained with an APC anti-human CD274 (B7-H1, PD-L1) antibody (Biolegend, San Diego, CA, USA) and a PE anti-human HLA-A,B,C antibody (Biolegend, 311406). The stained cells were read using LSRII flow cytometry (BD) and data were calculated using FlowJo software (version 10.9).

### 2.5. Site-Directed Mutagenesis

The HA-Aurora-D274N and HA-Aurora-D274A constructs were created using the QuikChange II XL Site-Directed Mutagenesis Kit (Catalog #200521) from Agilent, Santa Clara, CA according to the provided manual. The primer sequences are shown below.

Primer sequences:

HA-Aurora-D274N_F: TACTGACCACCCAAAATTTGCAATTTTAAGCTCTCCAGCT;
HA-Aurora-D274N_R: AGCTGGAGAGCTTAAAATTGCAAATTTGGGTGGTCAGTA;
HA-Aurora-D274A_F: GTACTGACCACCCAAAAGCTGCAATTTTAAGCTCTCCAG;
HA-Aurora-D274A_R: CTGGAGAGCTTAAAATTGCAGCTTTTGGGTGGTCAGTAC.

### 2.6. Standard Western Blot and Protein Capillary Electrophoresis

Standard Western blotting involved lysing the cells using Laemmli buffer supplemented with 1X protease and phosphatase inhibitor cocktail from Thermo Fisher. The cell lysates were then separated on an SDS PAGE gel obtained from Invitrogen (NP0321BOX), followed by incubation with the specific protein of interest. The resulting blots were visualized using the Azure Imaging System C300 from Azure Biosystems. The following primary antibodies were used: PD-L1 (E1L3N^®^) (Cell Signaling Technology, Catalog #13684; 1:500), Aurora A/AIK (1G4) (CST 4718; 1:500), GSK3β (27C10) (CST 9315; 1:500), phospho-GSK3β (Ser9) (CST 5558S; 1:500), Anti-HLA Class 1 ABC antibody [EMR8-5] (Abcam ab70328, 1:500), and β-actin (Sigma Aldrich A1978, clone AC15; 1:5000). The standard Western blot employed the use of secondary antibodies: anti-rabbit IgG (H+L) with HRP from Thermo Fisher, and anti-mouse IgG (H+L) with HRP from Thermo Fisher. Capillary electrophoresis was used to analyze the protein samples, which were run on a cartridge and then detected using the Wes instrument according to the manufacturer’s guidelines. The following primary antibodies were used in the protein capillary electrophoresis: PD-L1 (E1L3N^®^) (Cell Signaling Technology 13684; 1:25), Aurora A/AIK (1G4) (CST 4718; 1:25), GSK3β (27C10) (CST 9315; 1:25), phospho-GSK3β (Ser9) (CST 5558S; 1:25), and Vinculin (Abcam ab129002; 1:500). The following secondary antibodies were used: anti-rabbit secondary HRP antibody (ProteinSimple 042-206).

### 2.7. Real-Time PCR Analysis

Total RNA was obtained using the miRNAeasy Mini Kit from QIAGEN and transcribed with the qScript™ cDNA SuperMix Kit from QuantaBio (101414–106). Real-time PCR analysis of gene expression was conducted on the samples using PerfeCTa^®^ SYBR^®^ Green FastMix^®^ Reaction Mixes from Quantabio (101414–276). All RT-PCR reactions were carried out in quadruplicate, and fold changes were determined based on 18S in the threshold cycle.

Primer sequences:

PD-L1(CD274) Human_F: TGCCGACTACAAGCGAATTACTG;
PD-L1(CD274) Human _R: CTGCTTGTCCAGATGACTTCGG.

### 2.8. Microarray and Subsequent Gene Set Enrichment Analysis

Microarray: total RNA was collected using the miRNAeasy Mini Kit from QIAGEN (217004), and it was subjected to the microarray and sequencing resource at Boston University. The experiments used in this study for GBM22 and GBM22AR were deposited at GEO: GSE152612.

### 2.9. Plasmid Transfection and Lentivirus Transduction

For transfection, HA-EV (10792), pCMV-HA-PD-L1 (121492), and pCMV-HA-PD-L1 3SA (121493) were purchased from Addgene (Watertown, MA, USA). For transduction, AURKA shRNAs (TRCN0000010533, TRCN0000000657, TRCN0000000656) were purchased from Sigma. The pCDH-CMV-MCS-EF1α-Puro plasmid (Catalog #CD510B-1) was acquired from System Biosciences. pCDH-puro/Flag PD-L1 WT (121446) was obtained from Addgene.

### 2.10. In Vivo Mouse Model

An orthotopic PDX model was used, with GBM cells intracranially injected 3 mm lateral to the bregma and 3 mm below in 6- to 8-week-old C57BL/6 mice. Treatments continued until the animals showed moribundity or neurologic deficits such as retardation, lethargy, or seizures. Survival analysis utilized Kaplan–Meier survival fractions and statistical significance was determined using a log-rank test. The drugs used included Alisertib (30 mg/kg) dissolved in DMSO, cremophor EL (Sigma, 61791–12–6), Ethyl Alcohol (Pharmco-Aaper (Bohemia, NY, USA), 200 Proof), and PBS in a ratio of 10:32:8:50 (*v*/*v*/*v*/*v*). Drugs were administered three times per week; InVivoPlus anti-mouse PD-1 (BP0033-2) from BioXcell was given every three days.

### 2.11. Immunohistochemistry (IHC)

To facilitate antigen retrieval and exposure prior to further immunohistochemical processing, the paraffin-embedded brain tumor tissue sections were rehydrated and incubated with proteinase K (Agilent DAKO) for 5 min at 37 degrees Celsius. The tissue sections underwent antigen retrieval through exposure to a citric acid buffer and subsequent heating. Thereafter, primary antibody labeling with PD-L1 (E1L3N®) (Cell Signaling Technology, Catalog #13684 ), or NK1.1 (Catalog #MA1-70100) from Thermo Fisher was performed for 1.5 h at room temperature. Following the initial incubation with the primary antibodies, the tissue sections were further processed by incubating them with a secondary antibody, specifically a horse anti-mouse IgG antibody, at a dilution of 1:200 for 30 min. This secondary antibody labeling step was then followed by an additional 30 min incubation with an ABC-Peroxidase solution, also at a dilution of 1:50, carried out at room temperature. To visualize the antibody-labeled target proteins, diaminobenzidine was utilized as a colorimetric substrate, and the slides were subsequently counterstained with Hematoxylin to provide contrast and enhance the visualization of the tissue structures under conventional light microscopy. Slides were scored for intensity and percentage of cell-positive ones.

### 2.12. Statistical Analysis

We used Prism version 10.9 (GraphPad, La Jolla, CA, USA) to perform statistical significance tests, including the two-tailed student’s *t*-test or ANOVA for multiple comparisons. A *p*-value of less than 0.05 was considered statistically significant.

## 3. Results

### 3.1. AURKA Influences the Immune-Regulating Pathways in PDX GBM Cells

**Figure 1:**
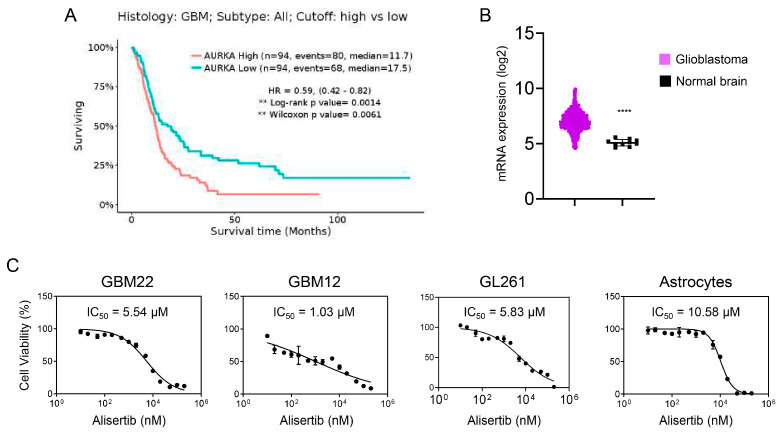
AURKA stands out as a promising therapeutic target in the context of GBM. (**A**) The survival curve of patients (wild-type and mutated IDH1) with different levels of AURKA mRNA in the GBM TCGA database is displayed. The cutoff point was determined using GlioVis through maximally selected rank statistics; (**B**) The TCGA GBM dataset was examined to assess the AURKA expression levels in both normal brain tissue and GBM tissue, revealing mRNA expression levels; (**C**) GBM22, GBM12, GL261, and astrocyte cells were subjected to escalating doses of Alisertib (ranging from 10 nM to 200 μM) for a period of 72 h, after which the viability of the cells was assessed (*n* = 4). Statistical significance was assessed by student’s *t*-test. Data are shown as mean ± SD. **** *p* < 0.001.

We aimed to investigate the potential connection between increased levels of AURKA mRNA and adverse prognosis among GBM patients through an analysis of the REMBRANDT database. Our findings suggested a correlation between elevated AURKA mRNA levels and poorer outcomes, highlighting the significance of this gene/protein as a key target for GBM treatment (Figure 1A). Furthermore, we noted increased expression of AURKA mRNA in tumors from GBM patients compared to normal brain tissue (Figure 1B). Increasing doses of the AURKA inhibitor Alisertib led to decreased cellular viability in both established and PDX GBM cells, confirming the critical roles of AURKA in GBM cell survival (Figure 1C). Notably, there was a milder impact on astrocytes, indicating a preference for targeting GBM cells.

**Figure 2:**
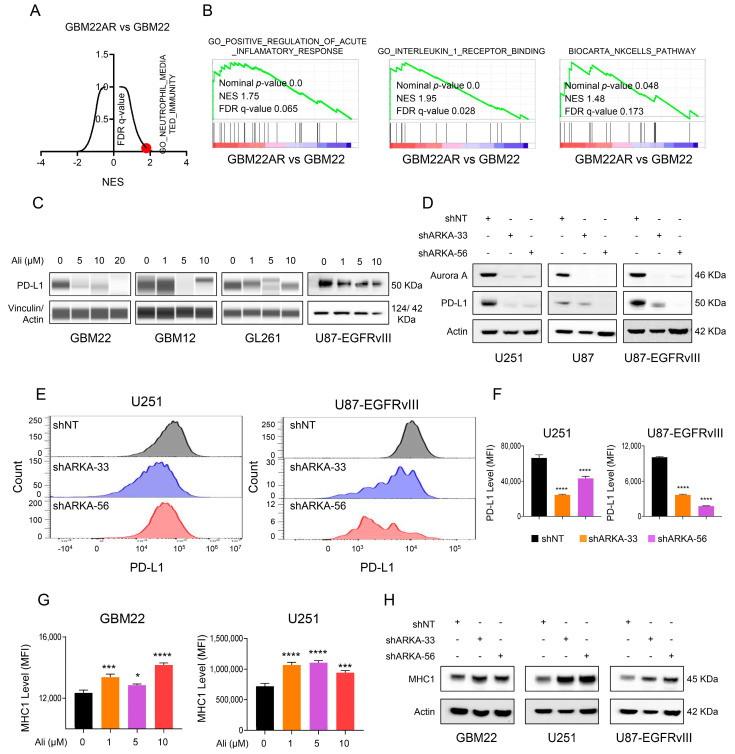
Inhibiting AURKA triggers an immune response in models of GBM. (**A**,**B**) Parental and chronically Alisertib-treated GBM22 cells underwent microarray analysis, followed by GSEA. An illustration of a volcano plot is presented in (A), where red dots highlight an increase in neutrophil-mediated immunity. Additionally, gene set enrichment analysis is depicted in (**B**). The data include normalized enrichment scores (NES) and FDR-q values for a sample size of 2; (**C**) Standard Western blot or protein capillary electrophoresis of GBM22, GBM12, GL261, and U87-EGFRvIII treated with increasing concentration of Alisertib for 24 h. Vinculin or actin is used as a loading control; (**D**) U251, U87, and U87-EGFRvIII cells were transduced with non-targeting shRNA or shARKA and the whole-cell protein lysates were subjected to standard Western blot; (**E**,**F**) U251 and U87-EGFRvIII cells were transduced with non-targeting shRNA or shARKA (shRNA) and labeled with PD-L1 antibody and analyzed by flow cytometry. The quantification is shown in F (*n* = 3); (**G**) Shown is the quantification of MHC1 level (MFI) of GBM22 and U251 cells treated with an increasing of Alisertib for 24 h; (**H**) GBM22, U251, and U87-EGFRvIII cells were transduced with non-targeting shRNA or shARKA and the whole-cell protein lysates were subjected to standard Western blot. Actin is used as a loading control. Statistical significance was assessed by ANOVA with Dunnett’s multiple comparison test. Data are shown as mean ± SD. * *p* < 0.05, ***/**** *p* < 0.001.

GBM is known for its resistance to apoptosis, which significantly affects the effectiveness of treatments involving temozolomide, radiation, and immune cells [8,30,31,32]. Understanding how tumor cells develop mechanisms to evade chemotherapy after prolonged drug exposure is crucial beyond acute treatment. Culturing cells with Alisertib for two weeks led to the generation of drug-resistant cells that exhibited partial resistance to Alisertib. Consequently, we conducted a transcriptome study on both parental GBM22 cells and those chronically treated with Alisertib. Our analysis of the data revealed that inhibiting AURKA had a significant impact on the immune system (Figure 2A,B). Our findings suggest that concurrently targeting the immune system and interfering with AURKA could be advantageous for GBM treatment. We proceeded to examine the potential influence of AURKA on immune checkpoint pathways, with a specific focus on PD-L1. This choice was informed by previous findings showing up-regulation of PD-L1 in GBM and its potential responsiveness to FDA-approved treatments [33]. Our research using various GBM model systems revealed that Alisertib had an effect on the expression levels of PD-L1. Initially, we observed a reduction in PD-L1 protein levels in PDX GBM models following exposure to Alisertib (Figure 2C). Subsequently, when testing the GL-261 model system, a murine GBM line widely used for assessing immune checkpoint inhibitor therapies in vivo, we noted a decrease in PD-L1 levels upon treatment with Alisertib (Figure 2C). Additionally, our analysis included U87 cells which are known to express relatively high levels of PD-L1, particularly within the context of EGFRvIII mutation. As expected, these cells exhibited robust baseline expression of PD-L1 which decreased upon exposure to Alisertib (Figure 2C).

Because kinase inhibitors may produce unintended effects, it is essential to ascertain whether Alisertib truly influences PD-L1 levels through its impact on AURKA. To address this, we carried out genetic loss of function experiments using shRNA. U251, U87, and U87 EGFRvIII GBM cells were transduced with lentiviral particles containing either non-targeting or two shRNAs against AURKA. Following selection, standard Western blotting of the harvested cells demonstrated reductions in both AURKA and PD-L1 protein levels (Figure 2D). Since PD-L1 is a protein found in the cell membrane, we conducted flow cytometry on U251 and U87 EGFRvIII GBM cells that were transduced with non-targeting or AURKA specific shRNA (Figure 2E,F). The results revealed a noticeable decrease in the levels of PD-L1 in the cell membrane, indicated by a significantly reduced MFI (Figure 2E,F). In addition to the PD-L1 immune checkpoint inhibitor molecule, we observed elevated surface expression levels of the MHC-class I molecule after administering Alisertib to GBM22 and U251 GBM cells (Figure 2G). Furthermore, introducing AURKA-specific shRNAs into GBM22, U251, and U87-EGFRvIII cells resulted in an increase in MHCI levels (Figure 2H). 

### 3.2. AURKA Regulates PD-L1 Levels Partly through GSK3β in a Posttranslational Manner

Next, we investigated how AURKA loss of function downregulates PD-L1. Previous studies have shown that the proteasome and the protein kinase GSK3β heavily regulate PD-L1 [34]. Specifically, phosphorylation of PD-L1 by kinase GSK3β leads to proteasomal degradation of PD-L1. Conversely, phosphorylation of GSK3β at Serine 9 inactivates the kinase, thereby stabilizing PD-L1 expression. This regulation of PD-L1 stability by GSK3β represents an important mechanism controlling immune checkpoint activation in the tumor microenvironment [34].

**Figure 3:**
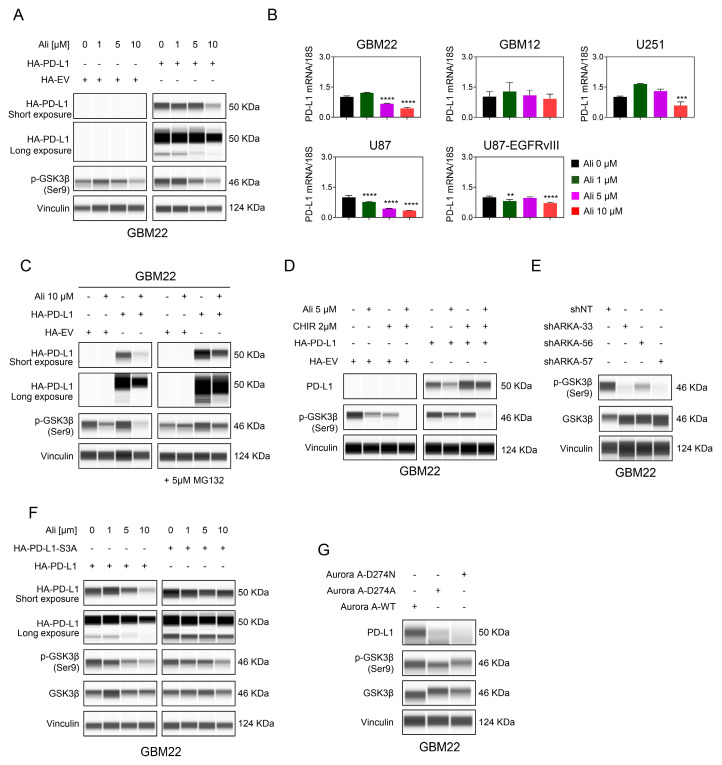
AURKA regulates PD-L1 levels in part through GSK3β in GBM cells. (**A**) The protein capillary electrophoresis of GBM22 cells transfected with HA-EV (empty vector) or HA-PD-L1 for 24 h and were treated with increasing concentrations of Alisertib for 24 h. Vinculin was used as a loading control; (**B**) Real-time PCR analysis of PD-L1 mRNA levels of GBM22, GBM12, U251, U87, and U87-EGFRvIII cells treated with increasing concentration of Alisertib for 24 h (*n* = 4); (**C**) The protein capillary electrophoresis of GBM22 cells transfected with HA-EV (empty vector) or HA-PD-L1 for 24 h and were treated with 10 µM Alisertib for 24 h in the presence or absence of 5 µM MG132; (**D**) The protein capillary electrophoresis of GBM22 cells transfected with HA-EV (empty vector) or HA-PD-L1 for 24 h and were treated with 5 µM Alisertib for 24 h in the presence or absence of 2 µM CHIR 99021, GSK3β inhibitor; (**E**) GBM22 cells were transduced with non-targeting shRNA or shARKA and the whole-cell protein lysates were subjected to protein capillary electrophoresis; (**F**) The protein capillary electrophoresis of GBM22 cells transfected with HA-PD-L1 or HA-PD-L1-S3A for 24 h and were treated with increasing concentration of Alisertib for 24 h; (**G**) The protein capillary electrophoresis of GBM22 cells transfected with Aurora A-D274N, Aurora A-D274A, or Aurora A WT. Statistical significance was assessed by ANOVA with Dunnett’s multiple comparison test. Data are shown as mean ± SD. ** *p* < 0.01, ***/**** *p* < 0.001.

In our study, GBM22 cells were transfected with a control or HA-tagged PD-L1 cDNA and then treated with increasing concentrations of Alisertib for analysis using capillary protein electrophoresis. Our results revealed that Alisertib reduced levels of HA-PD-L1 protein along with a decrease in phosphorylated GSK3β (Figure 3A), indicating posttranslational regulation of PD-L1 by Alisertib. Furthermore, we conducted real-time PCR analysis on several GBM cultures to measure PD-L1 mRNA levels following treatment with Alisertib. The results showed varied responses across different cultures. While GBM12 cells did not show significant transcriptional regulation of PD-L1, others such as the native U87 GBM line exhibited a more substantial response (Figure 3B). These findings suggest that multiple regulatory mechanisms are involved in modulating PD-L1 levels upon Alisertib treatment, encompassing both transcriptional and posttranslational regulation.

Our investigation aimed to evaluate whether Alisertib treatment triggers enhanced proteasomal degradation of PD-L1. To achieve this, we used MG132, a selective and potent inhibitor of the proteasome—a large multi-subunit protein complex responsible for degrading polyubiquitinated proteins within cells. By blocking the proteolytic activity of the proteasome, MG132 prevents the breakdown of target proteins tagged for destruction, such as PD-L1 and other regulatory proteins. This results in the stabilization, enhanced expression, and accumulation of PD-L1 and other key molecules within the cellular environment. In our study, GBM22 cells were transfected with a PD-L1 cDNA construct and then exposed to Alisertib, both in the presence and absence of MG132 (Figure 3C). As expected, treatment with MG132 reduced the Alisertib-induced decrease in PD-L1 levels. Upon observing a decline in phosphorylated GSK3β levels, we theorized that this kinase might play a role in reducing PD-L1 protein levels following exposure to Alisertib (Figure 3D). To investigate this hypothesis, GBM22 cells were transfected with a cDNA construct encoding PD-L1 and then treated with Alisertib alongside an inhibitor for GSK3β. Similar to the experiment involving the proteasomal inhibitor, inhibiting GSK3β partially reversed the reduction in PD-L1 levels caused by Alisertib (Figure 3D). To validate whether specific loss of function of AURKA activates GSK3β, protein capillary electrophoresis was performed to measure phosphorylated GSK3β levels. GBM22 cells expressing AURKA-targeting shRNA showed a decrease in phosphorylated GSK3β (Figure 3E). Previous research has demonstrated that GSK3β phosphorylates PD-L1 at three specific sites, which subsequently affects the stability of PD-L1 [34]. To investigate this further, GBM22 cells were transduced with wild-type PD-L1 or a non-phosphorylatable mutant protein (HA-PD-L1-3SA) and exposed to Alisertib (Figure 3F). Treatment with the aurora kinase inhibitor led to reduced levels of wild-type PD-L1, whereas its impact on the expression of phospho-mutant PD-L1 was much less pronounced (Figure 3F). This suggests that GSK3β likely plays a role in regulating PD-L1 when AURKA is blocked. Next, our aim was to investigate whether the AURKA’s kinase activity has an impact on PD-L1 protein levels. For this purpose, we introduced wild-type AURKA or kinase-deficient mutants into GBM22 cells. After conducting protein capillary electrophoresis, we observed that transfection with a kinase deficient mutant of AURKA resulted in a decrease in PD-L1 protein levels and phosphorylated GSK3β (Figure 3G). In summary, these results indicate that AURKA likely regulates PD-L1 levels through its kinase activity, which may affect the activity levels of GSK3β and subsequently influence PD-L1 protein levels.

### 3.3. Disrupting AURKA Can Enhance Immune Cells’ Capacity to Eliminate GBM Cultures

**Figure 4:**
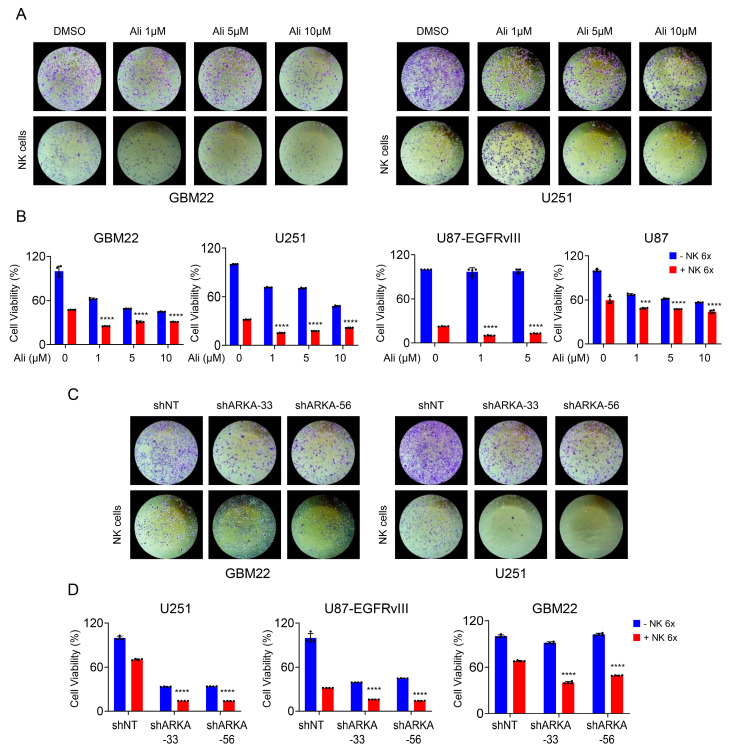
Loss of function of AURKA enhances NK-cell mediated reduction in cellular viability of GBM cultures. (**A**,**B**) GBM22, U251, U87-EGFRvIII, and U87 cells were treated with increasing concentrations of Alisertib for 48 h, and the NK-92 MI cells (6×) were added for another 24 h. Then, cellular viability was analyzed by Crystal Violet (*n* = 4). Quantification is shown in B; (**C**,**D**) GBM22 and U251 cells were transduced with non-targeting shRNA or shARKA and treated with NK-92 MI cells (6×) for 24 h. Then, cellular viability was analyzed by Crystal Violet (*n* = 4). Quantification is shown in D. Statistical significance was assessed by ANOVA with Dunnett’s multiple comparison test. Images were captured using a 4x objective lens. A statistically significant difference was observed between GBM cells treated with NK cells alone and GBM cells exposed to NK-92 MI cells in combination with varying concentrations of Alisertib. Data are shown as mean ± SD. ***/**** *p* < 0.001.

To investigate the impact of inhibiting AURKA on immune cell-mediated cytotoxicity, we utilized a co-culture model consisting of NK-92 MI cells and various GBM cells. Our analysis demonstrated the ability of NK-92 MI cells to diminish the viability of GBM22, U251, U87, and U87-EGFRvIII GBM cells, indicating susceptibility to NK-cell mediated suppression of tumor growth (Figure 4A,B). This augmentation was more pronounced in GBM22, U251, and U87 EGFRvIII cells compared to conventional culture with U87 (Figure 4A,B). To address specificity, three GBM cultures were transduced with either non-targeting shRNA or two specific shRNAs targeting AURKA. The enhanced NK-cell-mediated killing of GBM cells upon loss of AURKA is consistent with the effects of Alisertib (Figure 4C,D). 

**Figure 5:**
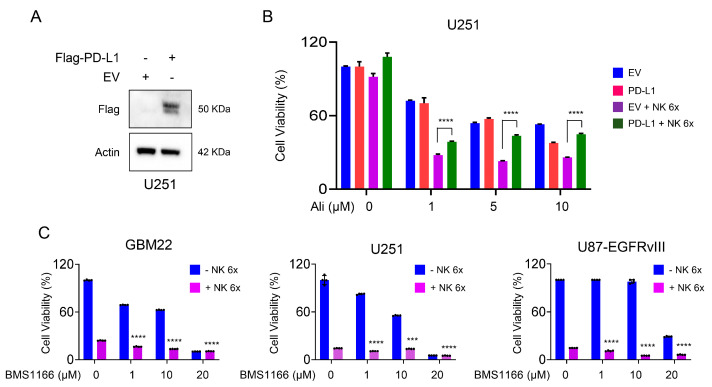
PD-L1 inhibitor reduces GBM cell viability but has minimal effect on NK-92 MI cell-mediated killing. (**A**) Stable cell lines of empty vector or Flag-PD-L1 over-expressed U251 cells were treated increasing concentrations of Alisertib for 48 h and the NK-92 MI cells (6×) were added for another 24 h. Shown is the quantification of cellular viability of Crystal Violet (*n* = 4); (**B**) Western blot of stable cell lines of empty vector or Flag-PD-L1 over-expressed U251 cells. Actin is a loading control; (**C**) GBM22, U251, and U87-EGFRvIII cell lines were exposed to escalating doses of BMS1166 for a period of 48 h, followed by the addition of NK-92 MI cells (6×) for an additional 24 h. Subsequently, cellular viability was assessed using Crystal Violet staining. The results present the quantification of cellular viability as determined by Crystal Violet assay. Statistical significance was assessed by ANOVA with Dunnett’s multiple comparison test. A statistically significant difference was noted between GBM cells treated with NK cells alone versus GBM cells exposed to NK-92 MI cells in combination with diverse concentrations of BMS1166. Data are shown as mean ± SD. ***/**** *p* < 0.001.

Since the reduction in PD-L1 was observed due to the loss of function of AURKA in GBM cells, we aimed to investigate whether PD-L1 loss plays a role in NK cell-mediated killing of GBM cells following inhibition of AURKA. For this purpose, U251 was transduced with a control vector or a plasmid encoding PD-L1, resulting in substantial ectopic expression of PD-L1 (Figure 5A,B). Subsequently, GBM cells were seeded and treated with vehicle or Alisertib in the presence or absence of NK-92 MI cells. We observed that increased expression of PD-L1 seemed to improve the effectiveness of Alisertib in reducing cellular viability (in the absence of NK92 MI cells (Figure 5A), but only at the highest concentration. However, when NK-92 MI cells were present, the enforced expression of PD-L1 on U251 cells counteracted the killing effect of NK-92 MI cells both with and without Alisertib (Figure 5A). These findings suggest that PD-L1 plays a role in enhancing the ability of Alisertib to facilitate NK-92 MI cell-mediated killing of U251 cells. In addition, we employed the PD-L1 inhibitor BMS1166 to investigate its potential in augmenting NK-92 MI cell-induced killing of GBM cells (Figure 5C). Our findings revealed that while BMS1166 independently reduced GBM cell viability, its impact on NK-92 MI cell-mediated killing of GBM cells was minimal (Figure 5C).

### 3.4. Alisertib Impacted the Microenvironment in the Syngeneic Mouse Model of GBM

**Figure 6:**
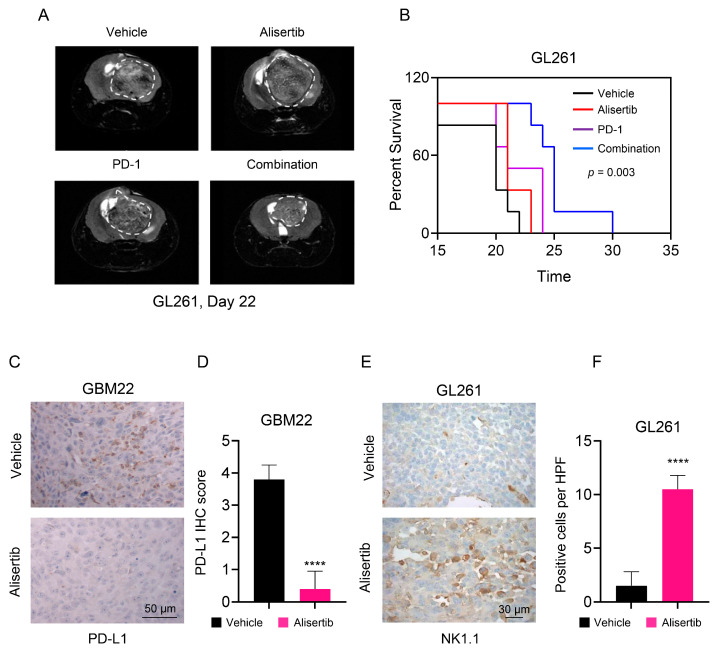
Alisertib impacted the microenvironment in the syngeneic mouse model of GBM. (**A**,**B**) GL261 cells were implanted in the right striatum of C57BL/6 mice. Four groups were randomly assigned: vehicle, Alisertib, PD-L1, and a combination of both, three days after the implantation. Mice were treated with Alisertib three times per week and anti-PD-1 every three days. The representative MRI image from each group after 22 days of implantation is shown in A and animal survival is provided in B (Kaplan−Meier-curve). The white dotted lines indicates the location of the tumor; (**C**,**D**) Tumors from a previously conducted GBM22 orthotopic xenograft experiment were fixed and stained with PD-L1 antibody. Quantification is shown in D. Scale bar: 50 µM; (**E**,**F**) Tumors from A were fixed and stained with antibody-detecting NK-cells (NK1.1). Quantification is shown in F. Scale bar: 30 µM. Statistical significance was assessed by student’s t-test. Data are shown as mean ± SD. **** *p* < 0.001.

Continuing our investigation, we proceeded to evaluate the potential enhancement of immune checkpoint inhibitor therapy by adding Alisertib using a mouse model. Our previous studies demonstrated Alisertib’s efficacy against glioma in PDX models [14]. We opted for the GL-261 syngeneic GBM mouse model, known for its partial responsiveness to immune checkpoint inhibitor therapy. In this model, GL-261 cells were implanted into the right striatum of immunocompetent mice. Subsequently, we established four groups: vehicle, anti-PD-1 alone, Alisertib alone, and a combination of both treatments (Figure 6A,B). Consistent with prior research from our lab and others, mice treated with the vehicle exhibited rapid deterioration due to aggressive GBM growth. While individual administration of Alisertib or anti-PD-1 did not yield significant therapeutic benefits, the concurrent use of both Alisertib and anti-PD-1 resulted in increased overall survival (Figure 6B). MRI scans of selected subjects from each group revealed smaller tumors in the combined treatment group compared to the control or single-treatment groups (Figure 6A).

Following Alisertib treatment in a previous orthotopic xenograft model, we analyzed PD-L1 expression. As expected, high PD-L1 levels were observed in GBM22 xenograft tumors treated with the vehicle. However, exposure to Alisertib led to reduced PD-L1 levels (Figure 6C,D), corroborating our earlier in vitro findings. Considering our observed enhancement in NK-cell mediated elimination of GBM cells in the lab, we speculated about the potential impact of AURKA inhibition on NK-cell infiltration in gliomas. Our results showed that Alisertib increased the quantity of NK cells within tumors compared to those treated with the vehicle (Figure 6E,F). These outcomes suggest that Alisertib might induce a partial reversal of the highly immune-suppressive GBM environment.

In conclusion, these findings highlight AURKA as a promising target for GBM therapy, especially in combination treatments aimed at modulating immune responses and creating an unfavorable tumor environment.

## 4. Discussion

Targeting aurora kinases continues to be a promising treatment approach for various types of cancer, such as breast cancer, lung cancer, and brain cancer [1,2,3,14,15,16,35,36]. This strategy is viable because inhibitors have been developed to target both AURKA (e.g., Alisertib) and aurora kinase B (barasertib). These compounds are also able to cross the blood–brain barrier and have demonstrated effectiveness when used alone in mouse models with GBM [1]. Alisertib has undergone clinical trials for GBM but, like other compounds, single-agent treatments typically do not yield significant results in patients. Additionally, many GBM trial patients have already received standard care before participating in clinical trials, making their tumors even more challenging to treat afterwards. It is known that this type of therapy causes a shift in the gene expression profile of GBM cells towards the mesenchymal phenotype [37,38]. Due to this reason, targeting AURKA may be particularly beneficial within combination drug therapies involving immunotherapy or new immune cell treatments.

GBM is often categorized as a “cold tumor” with limited T- or NK-cell infiltration and an immune-suppressive microenvironment containing M2 macrophages and/or MDSCs [4,7,39]. Furthermore, a portion of GBMs express PD-L1, contributing to the evasion of the immune system by GBM. Our results indicate that AURKA, overexpressed in GBM, may drive the immune-suppressive environment within GBM and its associated microenvironment. We presented multiple pieces of evidence supporting this claim. We found that AURKA seemed to induce PD-L1 expression in various GBM cell lines. This influence was partially reliant on GSK3β known to interact with AURKA [36] and occurred through posttranslational mechanisms. Previous research has indicated that GSK3β is implicated in the interaction with PD-L1 in different model systems and phosphorylates it at particular locations, influencing its stability. This process involves enzyme β-TrCP, an E3 ubiquitin ligase [34].

Another factor that is recognized for controlling the expression of PD-L1 is transcription factor c-Myc. Interestingly, GSK3β regulates c-Myc itself, and our previous research has connected AURKA activity to c-Myc levels in a GSK3β-dependent manner in model systems of glioblastoma, involving both adult and pediatric model systems [14]. Our earlier findings demonstrated that AURKA stimulates glycolysis and inhibits cellular respiration in a way that relies on both GSK3β and c-Myc. Previous studies revealed that glucose levels heavily regulate PD-L1 levels with depriving cancer cells of glucose specifically leading to reduced membranous PD-L1 levels [40]. Importantly, this effect was also induced by a ketogenic diet in mice, suggesting clinical significance [40].

Glucose deprivation caused the activation of AMPK, an energy-sensing kinase, which resulted in the phosphorylation and subsequent degradation of PD-L1 [40]. Furthermore, AMPK influenced the EZH2 pathway to promote gene expression associated with antigen presentation [40]. Previous research linking metabolism with PD-L1 signaling found that the anti-diabetic drug metformin was connected to this process. In addition to its various targets, sophisticated rescue experiments robustly demonstrated that metformin induces cancer cell death by inhibiting complex I of the respiratory chain. As a result, exposure of cancer cells to metformin leads to significant activation and phosphorylation of AMPK [40]. However, our previous studies did not show any activation of AMPK when AURKA inhibition was disrupted; therefore, we chose not to explore the connection between metabolism and PD-L1 levels in relation to modulation of AURKA. Metformin enhanced immune cell-mediated killing of cancer cells in vitro [40], consistent with our observation that Alisertib or genetic loss of function led NK cells effectively targeting GBM cells due to decreased levels of PD-L1 resulting from blocking AURKA.

Finally, we employed a syngeneic mouse model of glioma to show that Alisertib increases the effectiveness of immune checkpoint inhibitor treatment. We chose to use the GL261 model system as it exhibits some susceptibility to anti-PD-1 therapy and even greater susceptibility to the combination of anti-PD-1 and anti-CTLA4 therapy [33]. Considering that single-agent anti-PD-1 therapy is generally less effective than combined immune checkpoint inhibitor therapies, we tested whether adding the clinically validated AURKA inhibitor, Alisertib, would enhance overall survival. Our results revealed that combining Alisertib with anti-PD-1 therapy improved survival compared to using either treatment alone. This observation aligns with earlier findings from other researchers indicating that compounds influencing PD-L1 levels can boost the efficacy of immune checkpoint inhibitor therapy [34,40]. Gefitinib, for instance, demonstrated a capacity to decrease PD-L1 levels by depending on GSK3β, which in turn improved the effectiveness of anti-PD-1 therapy in mice [34]. We acknowledge that we did not determine PD-L1 levels in the GL261 model following the various treatments, as our current focus was on evaluating the effects of Alisertib treatment alone and not the various combination therapies at this stage of our studies. While this was a limitation of the current investigation, analyzing the impact of Alisertib on PD-L1 expression in this glioblastoma model and further assessing the effects of AURKA inhibition in combination with PD-1/PD-L1 blockade is an important next step to more comprehensively elucidate the complex relationship between these critical signaling pathways in the context of this highly aggressive brain cancer. Examining these relationships in greater depth will provide valuable insights that could guide the development of more effective combination treatment strategies targeting both the AURKA kinase and the immune checkpoint axis for glioblastoma patients. 

The relationship between AURKA inhibition and PD-L1 expression has been explored in multiple recent studies. While some investigations have found a positive association between AURKA expression or activity and PD-L1 levels [41,42], suggesting that AURKA may regulate PD-L1 expression, other studies have reported the opposite trend [43], indicating a more complex relationship between these two molecular players. These discrepancies in the observed relationship between AURKA and PD-L1 may arise from differences in experimental systems, tumor types, and specific mechanistic details that warrant further in-depth investigation to fully elucidate the underlying mechanisms. Ultimately, the interplay between AURKA and the PD-L1/PD-1 signaling axis appears to be complex and highly context-dependent, underscoring the importance of studying these relationships in diverse preclinical models and clinical settings to fully understand the therapeutic implications and potential for targeting these pathways in cancer treatment. Prior research has indicated the possible effectiveness of combining the AURKA inhibitor Alisertib with immune checkpoint inhibitors in other tumor types, such as breast cancer. This finding is consistent with the results of our current study using the GL261 syngeneic mouse model of glioblastoma. These data collectively suggest that a therapeutic strategy targeting both the AURKA kinase, and the immune checkpoint pathway could represent a promising approach for treating this highly aggressive and difficult-to-treat brain cancer. By simultaneously disrupting the immunosuppressive tumor microenvironment, this combination approach may offer improved clinical outcomes compared to targeting either pathway alone. 

## 5. Conclusions

GBM tumors frequently establish an immunosuppressive tumor microenvironment, but the underlying molecular and signaling mechanisms are not fully elucidated. This study supports the novel concept that the serine/threonine kinase AURKA is involved in this process. We demonstrate that AURKA can induce the expression of immune checkpoint protein PD-L1, thereby facilitating tumor immune evasion and suppressing the anti-tumor immune response. Targeting AURKA represents a promising cancer treatment strategy for brain tumors, particularly due to small molecule inhibitors like Alisertib that can penetrate the blood–brain barrier. However, Alisertib monotherapy has not shown significant clinical efficacy in glioblastoma patients. Combining AURKA inhibition with immunotherapies such as checkpoint blockade or novel immune cell-based treatments may offer improved therapeutic outcomes by simultaneously disrupting the immunosuppressive tumor microenvironment. Our research identifies AURKA as a key regulator of the immune response in GBM models, highlighting the potential for targeting this kinase in conjunction with immune-based therapies as a promising combination treatment strategy for this highly aggressive and difficult-to-treat brain cancer.

## Data Availability

The transcriptome data associated with this study are available at GEO: GSE152612. All other data are available from the authors upon reasonable request.
